# The oxygenating constituent of 3,6-diketocamphane monooxygenase from the CAM plasmid of *Pseudomonas putida*: the first crystal structure of a type II Baeyer–Villiger monooxygenase

**DOI:** 10.1107/S1399004715017939

**Published:** 2015-10-31

**Authors:** Michail N. Isupov, Ewald Schröder, Robert P. Gibson, Jean Beecher, Giuliana Donadio, Vahid Saneei, Stephlina A. Dcunha, Emma J. McGhie, Christopher Sayer, Colin F. Davenport, Peter C. Lau, Yoshie Hasegawa, Hiroaki Iwaki, Maria Kadow, Kathleen Balke, Uwe T. Bornscheuer, Gleb Bourenkov, Jennifer A. Littlechild

**Affiliations:** a The Henry Wellcome Building for Biocatalysis, Biosciences, College of Life and Environmental Sciences, University of Exeter, Stocker Road, Exeter EX4 4QD, England; b Biotechnology Research Institute, National Research Council Canada, 6100 Royalmount Avenue, Montreal, QC H4P 2R2, Canada; c Department of Biotechnology, Faculty of Engineering, Kansai University, Japan; d Department of Biotechnology and Enzyme Catalysis, Institute of Biochemistry, Greifswald University, Felix-Hausdorff-Strasse 4, 17487 Greifswald, Germany; e European Molecular Biology Laboratory (EMBL), Hamburg Outstation, Notkestrasse 85, 22607 Hamburg, Germany

**Keywords:** FMN-dependent monooxygenase, protein structure, industrial biocatalysis

## Abstract

The three-dimensional structures of the native enzyme and the FMN complex of the overexpressed form of the oxygenating component of the type II Baeyer–Villiger 3,6-diketocamphane monooxygenase have been determined to 1.9 Å resolution. The structure of this dimeric FMN-dependent enzyme, which is encoded on the large CAM plasmid of *Pseudomonas putida*, has been solved by a combination of multiple anomalous dispersion from a bromine crystal soak and molecular replacement using a bacterial luciferase model. The orientation of the isoalloxazine ring of the FMN cofactor in the active site of this TIM-barrel fold enzyme differs significantly from that previously observed in enzymes of the bacterial luciferase-like superfamily. The Ala77 residue is in a *cis* conformation and forms a β-bulge at the C-terminus of β-strand 3, which is a feature observed in many proteins of this superfamily.

## Abstract 2

The first crystal structure of a type II Baeyer–Villiger monooxygenase reveals a different ring orientation of its FMN cofactor compared with other related bacterial luciferase-family enzymes.

## 1. Introduction   

The use of enzymes in commercial biocatalysis is of increasing importance for the fine-chemical and pharmaceutical industries, with the number of new drugs produced in this way due to increase dramatically over the next few years (Wohlgemuth, 2010 ▸; Bornscheuer *et al.*, 2012 ▸; Reetz, 2013 ▸). The application of enzymes has considerable advantages in that the process is environmentally friendly and enzymes have the ability to produce single enantiomeric products, which are of value as chiral building blocks for new drug molecules (Lilly *et al.*, 1996 ▸; Littlechild *et al.*, 2007 ▸).

The Baeyer–Villiger oxidation reaction (Baeyer & Villiger, 1899 ▸) is an important reaction for synthetic organic chemistry in which linear/cyclic ketones react with peroxy acids to produce a corresponding ester/lactone. The application of the Baeyer–Villiger reaction for the chemical synthesis of antibiotics, steroids and pheromones has been reviewed by Krow (1993 ▸). The purity and stereospecificity of the reaction products required by the pharmaceutical industry is difficult to achieve by traditional chemical processes owing to the high reactivity of peracids (Roberts & Wan, 1998 ▸). The use of peracids and solvents on a large scale is not environmentally friendly. Therefore, there is increasing interest from industry in enzymes that can carry out a Baeyer–Villiger oxidation reaction (Alphand *et al.*, 2003 ▸).

Several microorganisms can produce NAD(P)H-dependent flavoenzymes capable of catalyzing Baeyer–Villiger reactions (Leisch *et al.*, 2011 ▸; Balke *et al.*, 2012 ▸), which use a stable flavin–peroxide intermediate to attack the carbonyl C atom of the substrate in a reaction identical to the equivalent non-enzymatic reaction. These enzymes are called Baeyer–Villiger monooxygenases (BVMOs) and are classified into two main types depending on the flavin cofactor. The more studied type I BVMOs are proteins utilizing the FAD cofactor, which is reduced by an NADPH bound to the same polypeptide chain. The first type I BVMO crystal structure was reported for the phenylacetone monooxygenase from *Thermobifida fusca* (Malito *et al.*, 2004 ▸). Type II BVMOs utilize a FMN cofactor, which is reduced by a separate NADH dehydrogenase enzyme. These enzymes have been relatively understudied compared with the type I enzymes owing to their more complex multi-enzyme structure. The substrate specificity of type II BVMOs is different from the type I enzymes and they are increasingly important for commercial biocatalysis since they have the ability to oxidize bicyclic lactones (Alphand *et al.*, 2003 ▸) and because they utilize the cheaper cofactor NADH.

**Figure 1:**
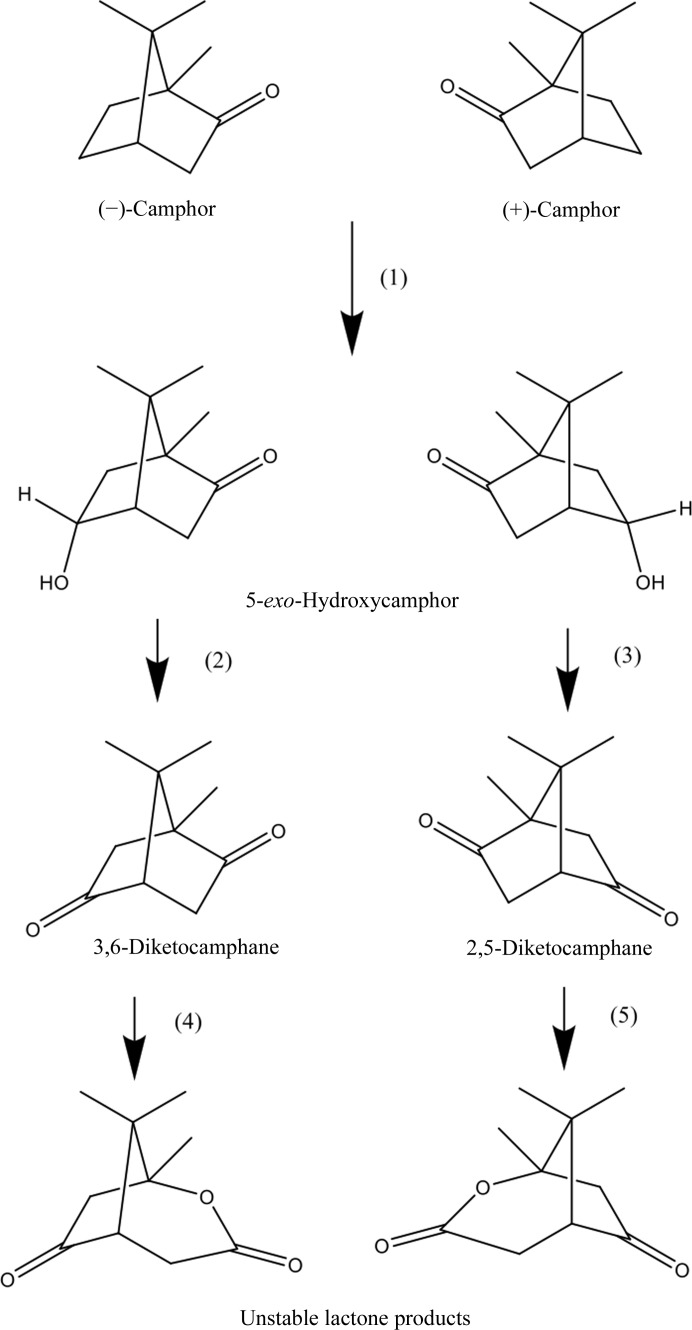
The camphor-degradation pathway (adapted from McGhie *et al.*, 1998 ▸). The following enzymes are indicated: (1) 5-*exo*-hydroxylase (P450_CAM_), (2) and (3) *exo*-hydroxycamphor dehydrogenases, (4) 3,6-DKMO, (5) 2,5-­DKMO.

It has been known for some time that the bacterium *Pseudomonas putida* NCIMB 10007 is able to grow on either enantiomer of the natural bicyclic monoterpene camphor as the sole carbon source (LeGall *et al.*, 1963 ▸). The oxidative degradation pathway of camphor requires two enantiocomplementary type II BMVOs (Conrad *et al.*, 1965 ▸), 2,5-diketocamphane 1,2-monooxygenase (2,5-DKMO; EC 1.14.13.162) and 3,6-diketocamphane 1,6-monooxygenase (3,6-DKMO; EC 1.14.13.–), which catalyse the NADH-dependent lactonization of diketocamphane stereoisomers (2,5- and 3,6-diketocamphane). The latter are intermediates in the oxidative degradation pathway of (+)- and (−)-camphor, respectively (Fig. 1 ▸). Both DKMOs are located on the large CAM plasmid harboured in *P. putida* and are induced when either racemic camphor or one of its stereoisomers are present in the growth medium (Shaham *et al.*, 1973 ▸). The DKMO enzymes have been reported to be built up from a dimeric oxygenating component and a loosely associated NADH dehydrogenase (Jones *et al.*, 1993 ▸). Both the 2,5-DKMO and 3,6-DKMO oxygenating components have sequence similarity to bacterial luciferases (McGhie, 1998 ▸) and bear little similarity to type I BVMOs. The molecular masses of the 2,5-DKMO and 3,6-DKMO monomers are 40.7 and 42.3 kDa, respectively (McGhie *et al.*, 1998 ▸).

The two DKMO enzymes have been shown to carry out oxy­genation reactions with a variety of natural ketones and their synthetic analogues using partially purified preparations of the wild-type enzyme (Grogan, Roberts & Willetts, 1993 ▸; Grogan, Roberts, Wan *et al.*, 1993 ▸; Grogan *et al.*, 1994 ▸; Gagnon *et al.*, 1994 ▸, 1995 ▸), where a mixture of both DKMOs was called MO1. This preparation was also able to utilize a range of aryl alkyl and alkyl alkyl sulfides. The two DKMOs can use natural and synthetic ketones with distinct enantioselectivity and they have been exploited in key reactions for the chemoenzymatic synthesis of commercially useful products (Grogan *et al.*, 1992 ▸; Grogan, Roberts & Willetts, 1993 ▸; Grogan, Roberts, Wan *et al.*, 1993 ▸; Gagnon *et al.*, 1994 ▸, 1995 ▸). The results of the different ketone and sulfide biotransformations were used to produce simple models for the substrate-binding pockets of both enzymes, which implied that there were differences between them (Beecher & Willetts, 1998 ▸).

The mechanism of enantioselectivity for the hydroxyl–peroxide rearrangement taking place in the BVMO active site was proposed by Kelly (1996 ▸) to be owing to diastereofacial selection by the flavin cofactor. Type II BVMOs were attributed to the R group in relation to the *re*-face attachment of the hydroperoxide to the flavin coenzyme.

Initial structural characterization studies of 3,6-DKMO by our group were with the native enzyme purified from camphor-induced *P. putida* cell cultures. This was the only isozyme able to be purified in sufficient quantities for crystallization (McGhie *et al.*, 1998 ▸). Only preliminary structural studies have been reported by us to date for the type II enzymes (McGhie *et al.*, 1998 ▸; Isupov & Lebedev, 2008 ▸). Detailed structural and mechanistic comparisons of the two DKMO isozymes, which have 45% sequence identity (Kadow *et al.*, 2012 ▸), and knowledge of their active sites will enable rationalization of their substrate specificity and their potential optimization by site-directed mutagenesis to use new industrially relevant substrates.

Multiple attempts to overexpress 3,6-DKMO in *Escherichia coli* were unsuccessful until the application of the Takara chaperone system (Kadow *et al.*, 2012 ▸). Using 3,6-DKMO produced using this expression system has allowed further protein crystals to be grown of the oxygenating subunit in both the apo form and in the presence of the weakly bound FMN cofactor. This has allowed the location of the bound cofactor and a comparison between the native enzyme and cofactor-complex structures to be carried out.

## 2. Experimental   

### 2.1. Materials   

All reagents were obtained from Sigma–Aldrich, Buchs, Switzerland unless otherwise stated. The chromatography columns were obtained from GE Healthcare (Little Chalfont, England).

### 2.2. Enzyme purification   

The original purification of the native 3,6-DKMO from 20 l batches of camphor-induced cells of *P. putida* NCIMB 10007 was reported by us previously (McGhie *et al.*, 1998 ▸; McGhie & Littlechild, 1996 ▸). The cells were harvested by centrifugation and disrupted by sonication. After the removal of cell debris, an ammonium sulfate precipitation step was followed by dialysis in buffer *A* [20 m*M* KH_2_PO_4_/K_2_HPO_4_ pH 7.1, 6 m*M* β-mercaptoethanol, 0.1 m*M* EDTA, 10 µ*M* phenylmethyl­sulfonylfluoride (PMSF), 20 µ*M* benzamidine (BAM)]. The protein was applied onto a Fast Flow Q anion-exchange chromatography column and eluted with a linear gradient of 0–0.45 *M* potassium chloride in buffer *A*. After dialysis in buffer *A*, the protein was applied onto a Mono Q anion-exchange column and eluted with a linear gradient of 0–0.45 *M* potassium chloride in buffer *A*. This was followed by size-exclusion chromatography in buffer *A* containing 5% ammonium sulfate using a Superose 12 gel-filtration column. The fractions containing native 3,6-DKMO were monitored by NADH consumption at 340 nm and by analysis of the products of the reaction by gas chromatography (McGhie *et al.*, 1998 ▸).

Recombinant 3,6-DKMO was purified from *E. coli* BL21 (DE3) cells (Novagen, Darmstadt, Germany) harbouring the pET-28b (Novagen) vector with the 3,6-DKMO gene and the plasmid pGro7 of the Takara Chaperone Plasmid Set (Takara, Saint-Germain-en-Laye, France) grown in LB medium containing 30 µg ml^−1^ kanamycin and 30 µg ml^−1^ chloramphenicol at 37°C to an optical density at 600 nm of 0.5–0.6 (Kadow *et al.*, 2012 ▸). The culture was transferred to 20°C and the chaperones were induced by the addition of 2 mg ml^−1^ l-arabinose and incubated for 30 min. Protein expression was induced with 1 m*M* isopropyl β-d-1-thiogalactopyranoside for 4 h at 20°C. The cells were harvested by centrifugation at 20 000*g*. The cell paste from a 2 l culture was resuspended in buffer *B* (20 m*M* KH_2_PO_4_/K_2_HPO_4_ pH 7.1, 6 m*M* β-mercaptoethanol, 0.1 m*M* EDTA, 10 µ*M* PMSF) at a concentration of 10%(*w*/*v*). Sonication was carried out using a Soniprep 150 (MSE UK Ltd, Lower Sydenham, England) followed by centrifugation at 20 000*g* to remove precipitated protein and cell debris. The N-terminally hexahistidine-tagged protein was purified on a HiLoad nickel column using a linear gradient of 0–1 *M* imidazole in buffer *B*. The enzyme was further purified by gel filtration on a Superdex 200 gel-filtration column using buffer *B* containing 0.1 *M* NaCl.

### 2.3. Crystallization   

Crystals of native 3,6-DKMO were grown at room temperature by the vapour-diffusion technique using purified protein at 10 mg ml^−1^ mixed in equal volumes with 50 m*M* PIPES pH 6.5, 50% ammonium sulfate (McGhie *et al.*, 1998 ▸). These crystals were flash-cooled in liquid N_2_ after a short soak in a cryoprotectant consisting of 30%(*v*/*v*) glycerol, 50 m*M* PIPES pH 6.5, 55% ammonium sulfate.

The overexpressed 3,6-DKMO protein was concentrated using a 10 kDa membrane Vivaspin (Vivascience, Massachusetts, USA). Microbatch crystallization trials were set up with an Oryx 6 crystallization robot (Douglas Instruments, Hungerford, England) using the Morpheus and JCSG+ screens (Molecular Dimensions, Newmarket, England) and protein solution at 7 mg ml^−1^ containing 20 m*M* FMN, 5 m*M* NADH and 5 m*M* (−)-camphor. The droplet consisted of a 50:50 ratio of protein solution to precipitant ratio and was covered with a 50:50 mixture of silicone and paraffin oils. The best crystals were flash-cooled in liquid N_2_ after a 30 s soak in a cryoprotectant consisting of 100 m*M* HEPES pH 7.0, 30%(*v*/*v*) PEG 200, 16% PEG 3350, 100 m*M* NaCl, 20 m*M* FMN.

### 2.4. Data collection   

The native 3,6-DKMO data were collected to 1.9 Å resolution (Table 1 ▸) at 100 K at station BW7B at the EMBL Outstation at DESY, Hamburg, Germany using a MAR345 image-plate detector (MAR Research, Norderstedt, Germany). Three-wavelength MAD data sets were collected at the Br *K* edge for the crystal of the native enzyme, which was soaked for 1 min in a cryoprotectant consisting of 1 *M* NaBr for Br anomalous phasing (Dauter *et al.*, 2000 ▸). These data were collected at 100 K at station BW6 at the Max Planck Institute at DESY, Hamburg, Germany (Table 1 ▸) using a MAR165 CCD detector (MAR Research). The data were processed using the *DENZO* suite of programs (Otwinowski & Minor, 1997 ▸).

Data for the complex of overexpressed 3,6-DKMO with FMN were collected at 100 K on beamline I04-1 at the Diamond Synchrotron light source (Oxford, England) using a PILATUS detector (Dectris, Baden, Switzerland). The complex data were processed and the native apo data were reprocessed using *XDS* (Kabsch, 2010 ▸) in the *xia*2 (Winter *et al.*, 2013 ▸) pipeline. Further data and model manipulation was carried out using the *CCP*4 suite of programs (Winn *et al.*, 2011 ▸).

### 2.5. Structure determination   

The structure of native apo 3,6-DKMO was solved by a combination of MR, Br-soak MAD and density modification, as previously described in Isupov & Lebedev (2008 ▸). MR trials were performed using a synthetic α_2_ dimer of the bacterial luciferase from *Vibrio harveyi* (16% sequence identity; Fisher *et al.*, 1996 ▸). An exhaustive translation search in *MOLREP* (Vagin & Teplyakov, 2010 ▸) was conducted for all possible orientations of the dimeric polyalanine model (Lebedev *et al.*, 2008 ▸) in which the orientation of its molecular dyad coincided with orientation of the noncrystallographic twofold axis determined from the self-rotation function at φ = 78.4°, ψ = 40.8°, χ = 180.0°. A convincing translation-function solution for a single orientation was subjected to rigid-body refinement in *MOLREP* (Vagin *et al.*, 1998 ▸) and positional refinement in *REFMAC*5 (Murshudov *et al.*, 2011 ▸). The 23 Br sites were located in the anomalous difference Fourier synthesis using phases obtained by multi-crystal averaging with *DMMULTI* (Cowtan, 2010 ▸) and were used in MAD phasing with *MLPHARE* (Otwinowski, 1991 ▸). While anomalous substructure search programs such as *SHELXD* (Sheldrick, 2010 ▸) can often find the Br sites for structures of this size (see, for example, Vivoli *et al.*, 2014 ▸), the sites in the 3,6-DKMO anomalous data could not be located, probably owing to a weak signal caused by the higher mosaicity of the soaked crystals.

The phases from *DMMULTI* averaging were used in *REFMAC*5 phased refinement (Pannu *et al.*, 1998 ▸) to produce a good-quality map that allowed the building of an apo 3,6-DKMO atomic model in *O* (Jones *et al.*, 1991 ▸).

The structure of the FMN complex of the overexpressed 3,6-DKMO was solved by MR with the refined structure of the apoenzyme using *MOLREP* (Vagin & Teplyakov, 2010 ▸). The structure was refined by *REFMAC*5 and the model was rebuilt in *Coot* (Emsley *et al.*, 2010 ▸). *BUSTER* refinement (Bricogne *et al.*, 2015 ▸) was used for better positioning of the cofactor with partial occupancy in the enzyme active site. The figures were prepared using *PyMOL* (DeLano, 2002 ▸) and *CCP*4*mg* (McNicholas *et al.*, 2011 ▸).

## 3. Results and discussion   

### 3.1. Purification and crystallization   

The native 3,6-DKMO crystallized in space group *P*2_1_2_1_2_1_, with unit-cell parameters *a* = 55.0, *b* = 93.4, *c* = 162.0 Å. The crystals that were grown in 50 m*M* PIPES pH 6.5, 50% ammonium sulfate (McGhie *et al.*, 1998 ▸) contained two DKMO subunits in the asymmetric unit with a solvent content of 50%. The crystal of native protein used for Br anomalous phasing and for multi-crystal averaging belonged to the same space group, with the different unit-cell parameters *a* = 54.9, *b* = 93.3, *c* = 140.8 Å and with 42% solvent content. The use of the Takara overexpression system was successful in producing a good yield of soluble 3,6-DKMO protein in *E. coli* host cells. The protein was purified by nickel-affinity and gel-filtration chromatography. The best overexpressed protein crystals grew at 18°C from 100 m*M* HEPES pH 7.0, 20% PEG 3350 in the presence of 20 m*M* FMN, 5 m*M* NADH and 5 m*M* (−)-camphor. These crystals contained a dimeric 3,6-DKMO molecule in the asymmetric unit and belonged to space group *P*2_1_2_1_2_1_, with unit-cell parameters *a* = 72.8, *b* = 82.8, *c* = 149.9 Å and with 54% solvent content.

### 3.2. Quality of the models   

The native apo structure was refined to an *R*_cryst_ and *R*_free_ of 17.1 and 22.1%, respectively, for all data in the resolution range 20–1.9 Å without a σ cutoff (Table 1 ▸). The FMN complex was refined to an *R*_cryst_ and *R*_free_ of 18.5 and 22.1%, respectively, at 75–1.9 Å resolution. The following residues were not modelled owing to disorder: *A*1, *A*128–133, *A*176–180, *A*377–378, *B*1, *B*127–135 and *B*176–179 in the native structure and *A*126–135, *A*176–179, *A*377–378, *B*125–135 and *B*176–179 in the FMN-complex structure.

The native 3,6-DKMO model also contains 798 water molecules, several glycerol molecules, four sulfate ions and two PIPES buffer molecules. The FMN-complex structure contains two cofactor molecules, with the occupancy of the cofactor modelled at 0.6, and several polyethylene glycol molecules of variable length. The two subunits of the native structure were rebuilt independently. The root-mean-square deviation (r.m.s.d.) between C^α^ positions of the two subunits is 0.23 Å. The FMN complex structure has been refined with local NCS restraints implemented in *REFMAC*5 (Murshudov *et al.*, 2011 ▸). The r.m.s.d. between the C^α^ positions of the two subunits is 0.19 Å. Both models contain no Ramachandran outliers as identified by *PROCHECK* (Laskowski *et al.*, 1993 ▸). The overall *G*-factors used as a measure of the stereochemical quality of the model are 0.2 for the native structure and 0.1 for the FMN complex (*PROCHECK*) and are better than expected for the reported resolution. A total of 80 side chains in the native structure and 92 in the FMN complex structure were modelled with alternative conformations. The residues Pro11 and Ala77 are in the *cis* conformation. Approximately 43% of the 3,6-DKMO amino acids are in α-helices, 14% in β-sheets and 5% in 3_10_-helices.

### 3.3. Overall fold   

**Figure 2:**
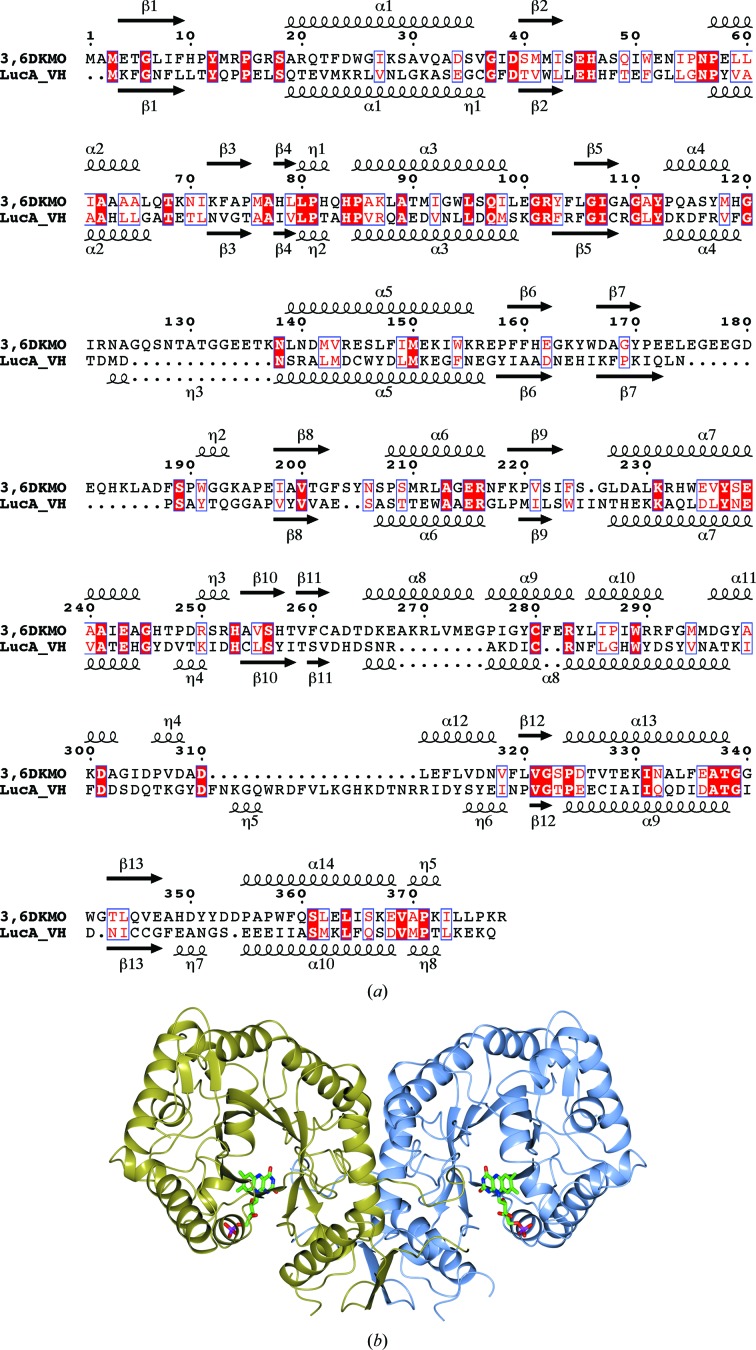
(*a*) Amino-acid sequence alignment of 3,6-DKMO with the α subunit of *V. harveyi* luciferase. The secondary-structure elements of 3,6-DKMO and luciferase are indicated above and below the alignment, respectively, as α-helices, η-helices (3_10_-helices) and β-strands. The secondary-structure assignment and the figure were produced using *ESPript* (Robert & Gouet, 2014 ▸). (*b*) A cartoon representation of the dimeric molecule of 3,6-DKMO showing its classical α/β-barrel structure. The FMN cofactor is shown as a stick model. Figs. 2(*b*), 4(*b*) and 5 were prepared using *CCP*4*mg* (McNicholas *et al.*, 2011 ▸)

The 3,6-DKMO monomer is made up from a single domain with an eight-stranded α/β-barrel (TIM-barrel) fold which has the nonprolyl *cis*-residue Ala77 forming a β-bulge in the middle of β-strand 3. The TIM-barrel structure (Banner *et al.*, 1976 ▸; Wierenga, 2001 ▸) is one of the most common protein folds, which can accommodate a variety of functions. Interestingly, according to the SCOP database (Andreeva *et al.*, 2014 ▸), these include at least two superfamilies of FMN-dependent oxygenases. These are the bacterial luciferase-like superfamily to which DKMOs belong (for example, luciferase from *V. harveyi*; PDB entry 1luc; Fisher *et al.*, 1996 ▸) and the FMN-linked oxidoreductase superfamily (for example, old yellow enzyme from *Thermus scotoductus* SA-01; PDB entry 3hgj; Opperman *et al.*, 2010 ▸). The 3,6-DKMO enzyme described in this paper contains two additional β-sheets: a hairpin formed by residues 159–162 and 167–170 and a parallel two-stranded β-sheet formed by residues 259–261 and 320–322 (Fig. 2 ▸ *a*).

Two 3,6-DKMO subunits join to form a dimeric molecule (Fig. 2 ▸ *b*) where approximately 3300 Å^2^ of solvent-accessible area is buried upon dimer formation (18% of the solvent-accessible area of a subunit).

### 3.4. FMN binding to DKMO   

In the course of the purification of the 3,6-DKMO enzyme the cofactor FMN is easily lost, especially if an ammonium sulfate precipitation step is used in the purification procedure, since the inorganic sulfate competes for the cofactor-binding site. We attribute the low affinity of the enzyme for the oxidized form of the cofactor to be owing to the catalytic cycle of DKMO. The enzyme is likely to have a higher affinity for the reduced form of the cofactor FMNH_2_ (FNR), which has a bent conformation in the isoalloxazine ring. Similarly, a 200-fold higher affinity for FNR compared with FMN has been reported for another two-component *p*-hydroxyphenyl­acetate monooxygenase from *Acinetobacter baumannii* (Sucharitakul *et al.*, 2006 ▸). The planar FMN, which has low affinity for the 3,6-DKMO active site, leaves it to be reduced to FNR by the FMN reductase. As the FMN reductase from the CAM plasmid could not be purified in significant amounts from *P. putida* preparations, commercially available NADH-FMN oxidoreductase from *V. harveyi* has successfully been used for reduction of the cofactor in activity measurements (McGhie, 1998 ▸). More recently, an *E. coli* flavin oxidoreductase was used to form a functional assembly with type II DKMOs (Kadow *et al.*, 2014 ▸).

### 3.5. FMN cofactor modelling in the electron density   

When the structures of unligated 3,6-DKMO and the α subunit of the holo bacterial luciferase (PDB entry 3fgc; Campbell *et al.*, 2009 ▸) are superimposed, the FMN ring of luciferase has steric clashes with the active-site residues of DKMO. It appears that either the isoalloxazine ring binds differently in the DKMO active site or some conformational changes need to occur to accommodate the FMN molecule in a similar orientation.

Multiple attempts to co-crystallize native DKMO and later the overexpressed protein with increasing concentrations of FMN were not very successful, as the low affinity of the oxidized cofactor for the 3,6-DKMO enzyme makes it difficult to achieve cofactor binding. No conformational changes were observed in the active site of FMN-complexed 3,6-DKMO, which is in agreement with the rigidity of the TIM-barrel scaffold. The best density for the cofactor molecule was achieved when the protein was co-crystallized in PEG with a large excess (20 m*M*) of FMN, the substrate analogue (−)-camphor and NADH. Attempts to use higher concentrations of cofactor resulted in the appearance of FMN crystals in the crystallization wells. A similarly poor affinity of the cofactor for the active site was observed in other reported structures of the bacterial luciferase-like fold superfamily enzymes which have either partial occupancy of FMN as in the *V. harveyi* luciferase (Campbell *et al.*, 2009 ▸) or have *B* factors which are significantly higher than those of neighbouring residues as observed in the structures of nitrilotriacetate monooxygenase from *Bacillus subtilis* and long-chain alkane monooxygenase from *Geobacillus thermodenitrificans* (PDB entries 1yw1 and 3b9o; Bonanno *et al.*, 2005 ▸; Li *et al.*, 2008 ▸).

**Figure 3:**
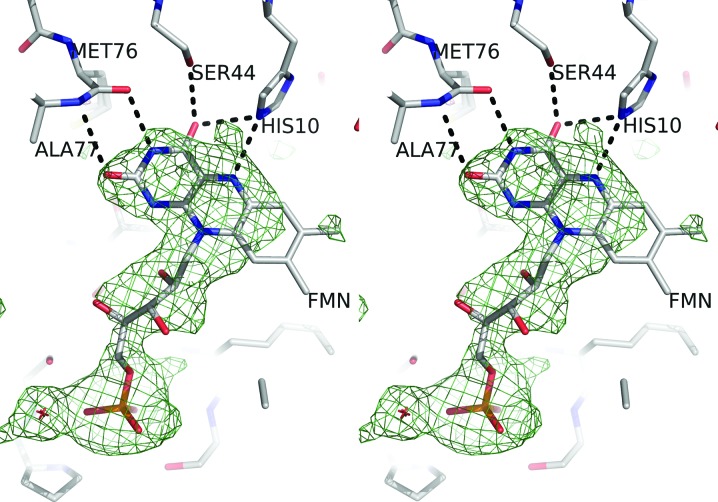
A stereo diagram of the active site of 3,6-DKMO viewed from the solvent region. The cofactor FMN, modelled with an occupancy of 0.6, and neighbouring residues are shown as stick models. The positive OMIT *F*_o_ − *F*_c_ electron-density map contoured at 3.0σ is shown in green and the negative *F*_o_ − *F*_c_ map contoured at 3.0σ is shown in red. The OMIT difference electron density was calculated by *BUSTER* using a structure from which all solvent and cofactor molecules had been removed and the model refined to reduce the bias. The figure was prepared using *PyMOL* (DeLano, 2002 ▸).

Therefore, detailed modelling of the position of the FMN cofactor in the 3,6-DKMO active site was carried out. When the native 3,6-DKMO model was positioned in the complex unit cell and refined by *BUSTER* (Bricogne *et al.*, 2015 ▸) prior to thee addition of solvent molecules, the weighted *F*_o_ − *F*_c_ electron-density maps clearly indicated the presence of the cofactor and the position of its phosphate group; however, the density for the isoalloxazine ring and the density for the ribityl sugar were less well defined (Fig. 3 ▸). The electron density clearly identifies the plane of the cofactor ring, with N5 of FMN hydrogen-bonded to His10. The cofactor atoms N3, O2 and O4 form hydrogen bonds to the 3,6-DKMO protein residues in this orientation. The isoalloxazine ring could also be modelled by a rotation of 180° around the C1′—N1 bond; however, in this case the C7M and C8M C atoms were seen to badly clash with the protein residues. Subsequent refinement varying the partial occupancy of the cofactor has indicated that a cofactor occupancy of 0.6 gives the best fit to both the 2*F*_o_ − *F*_c_ and *F*_o_ − *F*_c_ maps.

The poor electron density for FMN observed in the active site of 3,6-DKMO could indicate disorder related to the existence of multiple cofactor species created by photoreduction in the intense synchrotron X-ray beam. This has previously been reported for other flavin cofactor-containing enzymes (Røhr *et al.*, 2010 ▸). However, the density for the isoalloxazine ring of the cofactor in 3,6-DKMO appears to be flat and not butterfly-shaped as would be observed for the reduced species. We therefore consider the effect of photoreduction to be insignificant and attribute the poor electron density to the low affinity of the FMN for the active site of the enzyme.

This observation is supported by our experience with the structure of a related protein (Isupov & Littlechild, unpublished data), determined at 1.7 Å resolution, in which the FMN is at full occupancy in the active site. In this case the clear electron density shows that the FMN isoalloxazine ring is flat, thereby confirming the prevalence of the oxidized species. This reinforces our interpretation as described above for the 3,6-DKMO since crystals of both enzymes were subjected to a comparable radiation exposure.

No electron density was observed for the (−)-camphor or the NADH present in the crystallization medium.

### 3.6. The active site   

**Figure 4:**
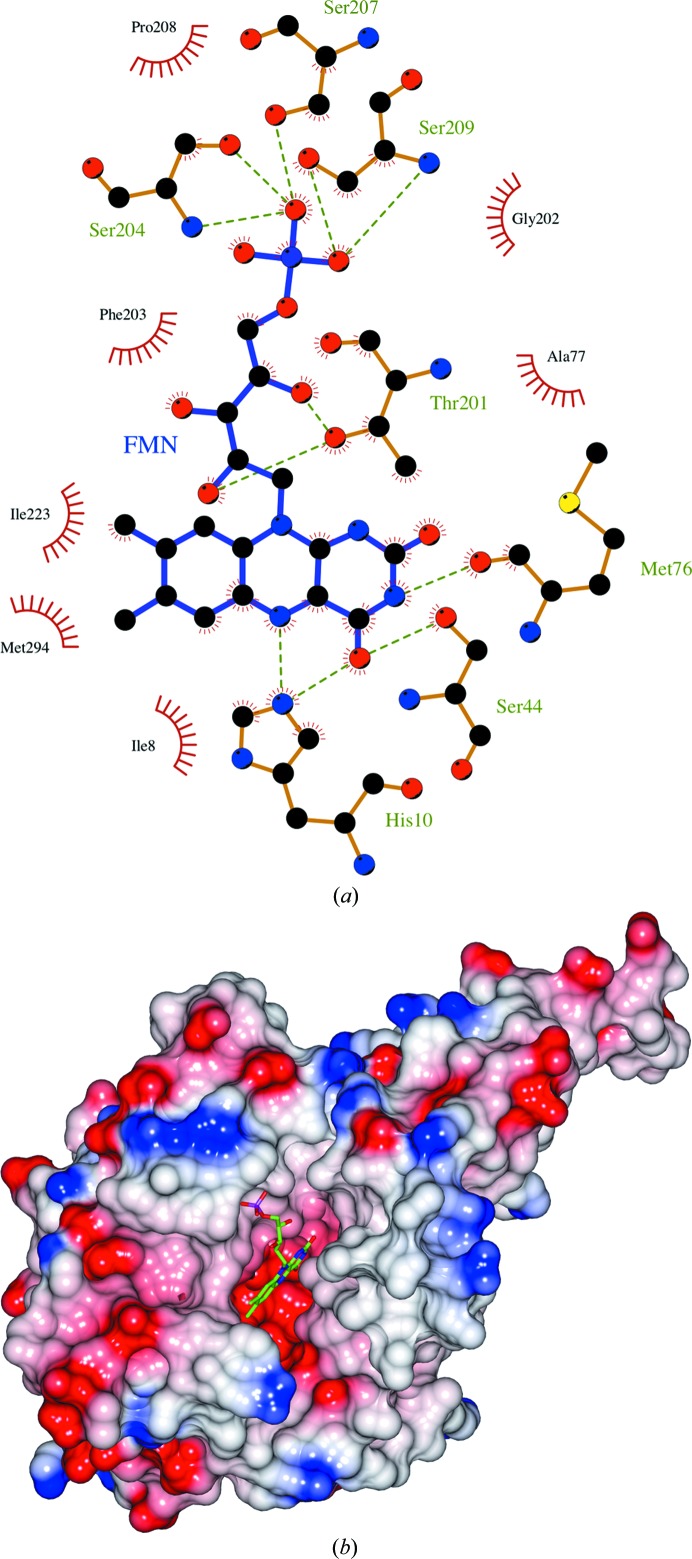
(*a*) A schematic drawing of the interactions of the FMN in the active site of 3,6-DKMO. The figure was prepared by *LigPlot*+ (Laskowski & Swindells, 2011 ▸). (*b*) The electrostatic potential surface calculated for the subunit of 3,6-DKMO. Positive charge is shown in blue and negative charge in red. The active-site cavity can be seen in the centre of the figure. The cofactor FMN molecule is shown as a stick model at the bottom of the active-site pocket.

The FMN cofactor binds in the cleft formed at the C-terminal side of the TIM barrel (Fig. 2 ▸ *b*), with the phosphate group involved in hydrogen bonding at the edge of the cleft and the isoalloxazine ring located deep inside of the cleft (Figs. 4 ▸ *a* and 4 ▸ *b*). The adjacent subunit contributes residues 175–180 to the lining of the active-site cavity on the opposite side of the cleft. This loop is poorly ordered in both the apo and the complex structures. The isoalloxazine ring makes hydrogen bonds to the side chains of His10 and Ser44 and the main-chain O atom of Met76.

The reduced form of the cofactor FMN (FNR) model was created using *JLigand* (Lebedev *et al.*, 2012 ▸), with the FNR isoalloxazine ring modelled with a 20° deviation from planarity, as observed in the structure of another luciferase-like fold superfamily enzyme, the F_420_-dependent secondary alcohol dehydrogenase enzyme (PDB entry 1rhc; Aufhammer *et al.*, 2004 ▸), and several other reduced flavin cofactor-containing structures. The FNR model was positioned into the active site of 3,6-DKMO, where it appears to retain the hydrogen bonding observed for the oxidized FMN (Fig. 4 ▸ *a*) but makes additional favourable hydrophobic interactions with the side chains of Phe203 and Ile223. These interactions would favour the higher affinity of the enzyme for the reduced form of the cofactor.

The catalytic reaction occurs on the *si* side of the isoalloxazine ring, which is consistent with the mechanism of other bacterial luciferase-like fold enzymes, but contradicts the predictions previously made for the *P. putida* type II BVMOs (Kelly, 1996 ▸).

### 3.7. Comparison with other bacterial luciferase-family proteins   

The dimer structure is conserved throughout the bacterial luciferase-like superfamily of TIM-barrel proteins and its conservation was used for macromolecular phasing of 3,6-DKMO (Isupov & Lebedev, 2008 ▸). Enzymes belonging to the other superfamily of TIM-barrel fold FMN-linked oxido­reductases also form dimers and occasionally higher oligomers, but these dimers are organized differently.

The closest structural homologue to 3,6-DKMO among known structures is subunit *A* of *V. harveyi* luciferase (PDB entry 1luc; Fisher *et al.*, 1996 ▸), which can be superimposed with the 3,6-DKMO structure with an r.m.s.d. of 2.2 Å for 292 C^α^ atoms of 362, as calculated by *Coot* (Emsley *et al.*, 2010 ▸). Superimposition of the 3,6-DKMO enzyme monomer C^α^ positions with the FMN-dependent long-chain alkane monooxygenase (PDB entry 3b9o; Li *et al.*, 2008 ▸) over 299 residues gives an estimated r.m.s.d. of 2.6 Å. The subunit of the F_420_-dependent secondary dehydrogenase enzyme (PDB entry 1rhc; Aufhammer *et al.*, 2004 ▸) can be superimposed with an r.m.s.d. of 2.8 Å over 280 residues.

**Figure 5:**
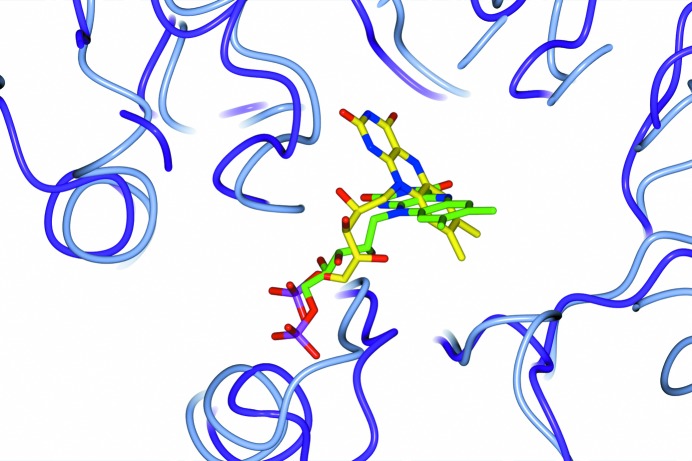
A diagram showing the different orientation of the isoalloxazine ring of the FMN cofactor in the active sites of the superimposed α subunit of the bacterial luciferase and the 3,6-DKMO enzyme. The C^α^ backbones of luciferase and 3,6-DKMO are shown in ice blue and purple, respectively. The C atoms in the FMN cofactors are shown in yellow and green for luciferase and 3,6-DKMO, respectively.

Known structures of FMN- and F_420_-dependent enzymes have a consistent orientation of their tricyclic cofactor rings within the clefts of their active sites (Aufhammer *et al.*, 2004 ▸, 2005 ▸; Bonanno *et al.*, 2005 ▸; Li *et al.*, 2008 ▸; Campbell *et al.*, 2009 ▸). The orientation of the isoalloxazine ring in the active site of 3,6-DKMO is nearly normal to the planes of the tricyclic cofactor rings observed in known structures of the bacterial luciferase-like fold superfamily enzymes, as illustrated in Fig. 5 ▸ for the bacterial luciferase α subunit. Despite this difference in the orientation of the cofactor ring, all enzymes of the bacterial luciferase superfamily catalyse their reaction on the *si* side of the ring.

### 3.8. The reaction mechanism   

The catalytic activity of the type II BMVOs is carried out mainly through the prosthetic FMN group. This cofactor binds in the enzyme active site in the FNR form, which undergoes attack by molecular oxygen at C4A of the isoalloxazine ring and becomes 4α-hydroperoxyflavin (FMN-OOH). This reaction is similar to that described for the flavin-containing monooxygenase from *Schizosaccharomyces pombe* (Eswara­moorthy *et al.*, 2006 ▸). The active-site residues are required for positioning of the substrate in a catalytic position in relation to the cofactor. Since there is a variation of the position of the cofactor isoalloxazine ring between 3,6-DKMO and the other proteins of the bacterial luciferase family, there is little conservation of the active-site residues within this enzyme family. Even the closely related 3,6- and 2,5-DKMOs have only two conserved residues lining the active-site cavity, which are His46 and Glu52 in 3,6-DKMO numbering. These are not conserved within the protein family. The residues His10 and Ser44 which are involved in FMN isoalloxazine ring binding in 3,6-DKMO are not conserved in 2,5-DKMO, implying a different mode of cofactor binding in this related enzyme.

### 3.9. Nonprolyl *cis*-peptide β-bulge   

A common feature of many bacterial luciferase-like fold superfamily enzymes is a nonprolyl *cis*-peptide forming a bulge in the middle of β3 which was proposed to be important for catalytic activity (Aufhammer *et al.*, 2005 ▸). This feature is not present in the nitrilotriacetate monooxygenase and long-chain alkane monooxygenase (Bonanno *et al.*, 2005 ▸; Li *et al.*, 2008 ▸). The importance of the nonprolyl *cis*-peptide β-bulge seems to be confirmed in the 3,6-DKMO structure, where this bulge is formed by residues Met76 and *cis* Ala77, with the main-chain O atom of Met76 and N atom of Ala77 contributing to the binding of the FMN ring.

### 3.10. Interaction with the flavin reductase   

The FMN reductase activity is required for the 3,6-DKMO reaction. When native 3,6-DKMO was purified from camphor-induced cells of *P. putida*, high enzymatic activity was observed in the crude extract (McGhie & Littlechild, 1996 ▸; McGhie *et al.*, 1998 ▸). This activity was present for the partially purified enzyme; however, it was lost in the later stages of purification of the oxygenating component. This led to the conclusion (McGhie *et al.*, 1998 ▸) that the oxygenating component forms a transient multienzyme complex with a specific *P. putida* flavin reductase. The commercially available *Vibrio* flavin reductase (Sigma–Aldrich) was able to demonstrate activity with the purified 3,6-DKMO oxygenating enzyme in biotransformation reactions (McGhie, 1998 ▸). More recently, it has been reported (Kadow *et al.*, 2014 ▸) that one of the flavin reductases present in *E. coli* is able to catalyse the FMN reduction with both 3,6- and 2,5-DKMOs. Partial sequencing of the large CAM plasmid has now identified a flavin reductase adjacent to the 3,6-DKMO gene on the CAM plasmid (Littlechild & Isupov, unpublished data), and studies are ongoing to reconstitute the putative multienzyme complex in a stable form to allow its structural characterization.

## Supplementary Material

PDB reference: type II Baeyer–Villiger monooxygenase, apo, 5aec

PDB reference: FMN complex, 4uwm

## Figures and Tables

**Table 1:** Summary of data-collection, phasing and refinement statistics Values in parentheses are for the outer resolution shell.

			Native bromide soak		
	FMN complex	Native	Peak†	Inflection†	Remote†
Data collection					
Space group	*P*2_1_2_1_2_1_	*P*2_1_2_1_2_1_	*P*2_1_2_1_2_1_		
Unit-cell parameters ()	*a* = 72.8, *b* = 82.8, *c* = 149.9	*a* = 55.0, *b* = 93.3, *c* = 161.9	*a* = 54.9, *b* = 93.3, *c* = 140.8		
*V*_M_ (^3^Da^1^)	2.67	2.46	2.13		
Beamline	I04-1, Diamond	BW7B, DESY	BW6, DESY		
Wavelength ()	0.9200	0.8443	0.9177	0.9201	0.8500
Resolution ()	55.601.90 (1.951.90)	20.441.93 (1.981.93)	13.002.70 (2.752.70)	13.002.75 (2.802.75)	13.002.45 (2.492.45)
Completeness (%)	99.5 (97.8)	98.9 (88.3)	99.1 (98.0)	99.3 (99.4)	97.1 (88.6)
*R*_merge_ ‡	0.085 (0.95)	0.127 (0.686)	0.087 (0.253)	0.091 (0.211)	0.113 (0.319)
*I*/(*I*)	14.9 (2.0)	9.9 (2.0)	14.9 (2.8)	17.2 (6.3)	9.5 (2.6)
Multiplicity	6.7 (6.8)	3.9 (3.4)	3.2	3.2	2.2
Wilson *B* factor (^2^)	34.7	27.5	33.9	34.5	37.8
Phasing					
No. of sites			14		
Phasing power§			1.28		
*R*_cullis_ ¶			0.72		
FOM††			0.26		
Refinement					
Resolution ()	55.601.90	10.441.93			
No. of reflections	71728	59253			
*R*_work_/*R*_free_ ‡‡ (%)	18.5/22.1	17.1/22.1			
Refined residues	725	728			
Ligand atoms	62				
Waters	616	798			
*B* factors§§ (^2^)					
Protein	33.0	26.0			
Ligand	55.1				
Water	42.2	33.9			
R.m.s deviations					
Bond lengths ()	0.009	0.010			
Bond angles ()	1.2	1.2			
Ramachandran plot analysis¶¶ (% of residues)					
Most favoured	91.0	90.1			
Additionally allowed	9.0	9.9			
Generously allowed	0	0.			
Disallowed	0	0			

† *I*(+) and *I*() were scaled separately for anomalous data.

‡ *R*_merge_ = 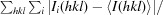 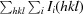, where *I*(*hkl*) is the intensity of reflection *hkl*, 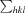 is the sum over all reflections and 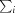 is the sum over *i* measurements of the reflection.

§ Phasing power = *F*_H_/*E*, where *E* is the estimated lack-of-closure error.

¶ *R*_cullis_ = lack-of-closure error/isomorphous difference and is quoted for centric reflections only.

†† FOM is the overall figure of merit, defined as the estimated cosine of the phase error.

‡‡ *R*_cryst_ = 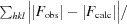 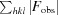.

§§ The Wilson *B* factor was estimated by *SFCHECK* (Vaguine *et al.*, 1999 ▸).

¶¶ Ramachandran plot analysis was performed by *PROCHECK* (Laskowski *et al.*, 1993 ▸).
